# The Role of the Toothbrush in Oral Hygiene

**Published:** 1922-04

**Authors:** Benjamin H. Rost

**Affiliations:** New York, N. Y.


### THE ROLE OF THE TOOTHBRUSH IN ORAL HYGIENE

BY BENJAMIN H. ROST, D.D.S., NEW YORK, N. Y.

The toothbrush, because of its practically universal use, ought to be one of the most important if not the most important aid in oral hygiene.

In searching into the etiology of gingival inflammations we find that calcareous deposits at the gingival margins of the teeth stand out most prominently, although other irritants of a chemic, septic and mechanic nature, such as strong drugs, micro-organisms and vigorous brushing, are not to be lost sight of.

Of the irritants above mentioned I have found that stiff-bristle brushes and strenuous brushing are the worst, producing a condition in the oral cavity which defeats the purpose of oral hygiene.

Burgess states that oral hygiene has for its goal a physiological status and includes ways and means to establish and maintain the vitality, tone and resistance of the teeth and tissues in the oral cavity.

I have come to the conclusion that the goal in oral hygiene will not be reached as long as our general practitioners and periodontists continue to advocate and advise brushing the gums and teeth with stiff-bristle toothbrushes.

Merritt avers that anything that irritates the gingivae is a potential cause of pyorrhea.

Bunting points out that the most vulnerable spot in the mucous membrane covering the mouth is to be found in the gingival tissues, where the protecting covering is easily and frequently broken and through which infection may most readily gain entrance to the deeper tissues.

Fones prefers a soft-bristle toothbrush, recognizing that the mucous membrane is thin and quite often damaged.

Adair advocates a soft-bristle brush for the reason that it is sufficient to clean and polish the surfaces of the teeth.

Bell warns against the use of cheap toothbrushes whose bristles break off and often lodge in the interproximal spaces and gingival trough.

Sarrazin believes the small toothbrush is best.

As to my own observations, although limited, I have found that the mucous membrane covering the gums is a delicate structure indeed and not strong enough to withstand the irritating effects of a stiff-bristle brush used from once to thrice daily. It is a fact that up to the present time the toothbrush has been a prolific cause of gingivitis and buccal abrasions, lacerating the fine mucous membrane covering the gums, thus preparing the soil for those diseases we are endeavoring to avoid. With a gingival inflammation produced we have established a *locus minoris resistentiae,* which allows ready penetration of pathogenic organisms into the deeper tissues.

Williams has given us the measurement from the points of the cuspids to the buccal groove of the first molars to be 23.5 mm. in the average dental arch. The average toothbrush found in the household has its rows of bristle tufts about 2 inches in length, which I have found to be at least 5/16 of an inch too long. When the anterior teeth are brushed the cuspids are included, and when the posterior teeth are brushed, again the cuspids are included. As a result of this excessive length the cuspids, together with the gums surrounding them, receive intensive brushing resulting in the devitalization of tissue. On careful inspection of the cuspid area one may readily observe in a very large number of cases denudation of the mucous membrane at the gingival borders and abrasion of the enamel of the tooth. To overcome this fault the toothbrush should conform to No. 2 in my outline of the ideal toothbrush below.

Harrison and others have proved that the commonly used toothbrush harbors enormous quantities of pathogenic bacteria. In view of these observations one can readily understand how far-reaching in its effects upon the vulnerable mucous membrane the septic toothbrush can be. The deleterious consequences may be overcome to a great extent by adopting an ideal toothbrush for universal use possessing the following features:

1. Soft bristles (preferably goat's hair).
2. Length of the bristle tufts not to exceed 1 3/16 inches.
3. Three parallel rows of bristle tufts.
4. Replaceable bristles, to be used once and discarded.
5. Space between the rows of tufts 1/32 inch.
6. Brushing surface should present a uniformly slightly concave surface both longitudinally and in width.
7. Handle may be made of amber, celluloid, ivory or bone.

It is my opinion that the toothbrush should under no circumstances be used for the purpose of massaging the gums. For obtaining vigorous circulation in the gums, massaging regularly with the clean index finger is very satisfactory.

Our duties toward our patients should not cease with the recommendation to "brush your teeth," but should consist of instructing them in the kind of a brush to employ and how to properly use it.—*Dental Cosmos*, February, 1922.

